# Dosage effects of organic manure on bacterial community assemblage and phosphorus transformation profiles in greenhouse soil

**DOI:** 10.3389/fmicb.2023.1188167

**Published:** 2023-05-02

**Authors:** Liangliang Zhang, Junfang Niu, Xuewei Lu, Ziyue Zhao, Kaixuan Li, Fenghua Wang, Chaochun Zhang, Ruibo Sun

**Affiliations:** ^1^Anhui Province Key Lab of Farmland Ecological Conservation and Pollution Prevention, Engineering and Technology Research Center of Intelligent Manufacture and Efficient Utilization of Green Phosphorus Fertilizer of Anhui Province, Research Centre of Phosphorous Efficient Utilization and Water Environment Protection Along the Yangtze River Economic Belt, College of Resources and Environment, Anhui Agricultural University, Hefei, China; ^2^Key Laboratory of JiangHuai Arable Land Resources Protection and Eco-restoration, Ministry of Natural Resources, Hefei, China; ^3^Key Laboratory of Agricultural Water Resources, Hebei Key Laboratory of Soil Ecology, Center for Agricultural Resources Research, Institute of Genetics and Developmental Biology, Chinese Academy of Sciences, Shijiazhuang, China; ^4^Hebei Key Laboratory of Environmental Change and Ecological Construction, Hebei Experimental Teaching Demonstrating Center of Geographical Science, School of Geographical Sciences, Hebei Normal University, Shijiazhuang, China; ^5^College of Resources and Environmental Sciences, National Academy of Agriculture Green Development, Key Laboratory of Plant-Soil Interactions, Ministry of Education, China Agricultural University, Beijing, China

**Keywords:** greenhouse soil, manure, microbial P transformation, inorganic P solubilization, organic P mineralization

## Abstract

Manure is a potential substitute for chemical phosphate fertilizer, especially in intensive agriculture, such as greenhouse farming, but the associations between soil phosphorus (P) availability and the soil microbial community under manure application instead of chemical phosphate fertilizers are still rarely addressed. In this study, a field experiment in greenhouse farming with manure application instead of chemical phosphate fertilizers was established, including five treatments: a control with conventional fertilization and chemical phosphate fertilizer substitution treatments using manure as the sole P resource at 25% (0.25 Po), 50% (0.50 Po), 75% (0.75 Po), and 100% (1.00 Po) of the control. Except for 1.00 Po, all the treatments applied with manure harbored similar levels of available P (AP) as the control. Most of the bacterial taxa involved in P transformation were enriched in manure treatments. Treatments of 0.25 Po and 0.50 Po significantly enhanced bacterial inorganic P (Pi) dissolution capacity, while 0.25 Po decreased bacterial organic P (Po) mineralization capacity. In contrast, the 0.75 Po and 1.00 Po treatments significantly decreased the bacterial Pi dissolution capacity and increased the Po mineralization capacity. Further analysis revealed that the changes in the bacterial community were significantly correlated with soil pH, total carbon (TC), total nitrogen (TN), and AP. These results revealed the dosage effect of the impact of manure on soil P availability and microbial P transformation capacity and emphasized that an appropriate dosage of organic manure is important in practical production.

## Introduction

As typical intensive agriculture, greenhouse farming is a potential approach to cope with food demand in future (Aznar-Sánchez et al., 2020). Greenhouse production has rapidly developed in the past several decades, especially in developing countries, such as China. Greenhouse farming supplies types of agricultural products, especially vegetables and fruits, which are a great complement to traditional agriculture. However, the high input in greenhouse farming also results in many environmental problems, such as heavy metal contamination and nutrient loss (Yang et al., 2014). Thus, controlling pollution and nutrient flows is key for the sustainability of greenhouse farming (Zikeli et al., 2017).

P is a macroelement for plants. To increase plant production, high amounts of P fertilizers are applied to the soil. As a result, approximately 80% of global phosphate rock is consumed as fertilizer (Van Vuuren et al., 2010). However, phosphate rock is a nonrenewable resource; thus, P shortages may become one of the greatest challenges for sustainable agriculture in future (Cordell et al., 2009).

Manure is a good substitute for mineral P fertilizers, as it is rich in P. A large amount of manure produced by livestock production largely changes the P cycle in agriculture (Bouwman et al., 2013). Integration of manure in crop production is beneficial to the reduction in nutrient flow. The P in manure is bioavailable; thus, manure directly increases the soil AP content by supplying a high amount of AP. In addition, manure also contains other types of matter, such as organic carbon, which indirectly impacts soil P availability by changing soil properties and microbial P transformation capacity. For example, manure application could decrease the AP content by neutralizing soil acidity, as some manures are alkaline (Sun et al., 2015b). The high content of organic carbon in manure also changes the soil resource supply, thus changing the soil microbial community, especially for heterotrophic microbes, such as fungi (Sun et al., 2016), and impacting microbial P transformation (Shen et al., 2011). In addition, the types of organic substances in manure change soil absorption and desorption features targeting P, resulting in changes in P dynamics in soil (Von Wandruszka, 2006).

Microbes are the primary drivers of P transformation in soil (Li et al., 2021). The microbial community and P transformation features are sensitive to agricultural fertilization (Dai et al., 2020). Our previous studies have found that manure incorporation greatly changes soil fungal community assemblages by altering soil characteristics, especially the soil carbon pool (Sun et al., 2016, 2020). Another study revealed that manure addition enhances soil microbial organic P mineralization by supplying labile carbon, which increases the abundance of microbial taxa involved in organic P mineralization (Chen et al., 2020). However, compared with chemical fertilizers, manure is considered a friendlier substrate for soil microbial ecology, as many studies have shown that manure is more beneficial to the stabilization of soil microbial assemblages (Sun et al., 2015a, 2023b); thus, using manure to substitute mineral fertilizers may also be conducive to improving soil microbial diversity and ecological functions. Our recent study showed that the substitution of manure for mineral P fertilizers significantly increased soil P availability and enhanced microbial organic P mineralization capacity (Sun et al., 2022a). The results confirmed the feasibility of applying manure instead of mineral P fertilizers in greenhouse farming. However, the high AP content may also increase the risk of P leaching. Thus, finding the optimal dosage of manure in substituting mineral P fertilizer to balance agricultural production and environmental effects is important. In addition, how manure impacts the soil microbial community and P transformation features is still unknown. In this study, the bacterial community assemblage and P transformation features under a series dosage of manure input were determined. This study aimed to (i) determine the dosage effect of manure on the soil bacterial community and P transformation features, (ii) reveal the key factors influencing the soil bacterial community assemblage, and (iii) explore the optimal dosage of manure for mineral P fertilizer substitution.

## Materials and methods

### Experimental design

The design of the experiment was detailed in a previous study (Sun et al., 2022a). The field experiment was performed in a solar greenhouse in Raoyang County, Hebei Province, China (38°15′N, 115°44′E). The soil was silt loam. A total of five treatments were set up in 2017, including one control (conventional fertilization) and four treatments using manure substitute mineral P fertilizers with total P inputs of 25% (0.25 Po), 50% (0.50 Po), 75% (0.75 Po), and 100% (1.00 Po). The control treatment was fertilized with mineral nitrogen (N, 90 kg N·ha^−1^·year^−1^), P (90 kg P_2_O_5_·ha^−1^·year^−1^), and potassium (K, 90 kg K_2_O·ha^−1^·year^−1^) fertilizers and cattle manure (84 t ha^−1^·year^−1^). The manure-amended treatments were fertilized with the same amount of mineral N and K fertilizers as the control but with manure instead of mineral P fertilizers. The amount of manure applied to the 0.25 Po, 0.50 Po, 0.75 Po, and 1.00 Po treatments was 33.6, 67.2, 100.8, and 134.4 t ha^−1^·year^−1^, respectively. Each treatment contained three replicate plots. The size of each plot was 20 m^2^ (8 m × 2.5 m). The plant system in all treatments was tomato and muskmelon rotation. Except for the fertilization strategy, all the treatments were under the same management.

### Soil sampling, DNA extraction, PCR, and high-throughput sequencing

Soil sampling and soil bacterial community measurement were performed according to the methods described in previous studies (Sun et al., 2022; Sun et al., 2023a). In total, five soil cores (0–20 cm) were collected from each plot on 10 June 2020. They were mixed thoroughly as a single sample. After sieving through a sifter (bore diameter of 2 mm) to remove impurities, all the samples were divided into two parts: one for physicochemical property measurement and the other for DNA extraction.

A FastDNA Spin Kit for Soil (MP Biomedicals, Santa Ana, CA, the United States) was used for soil total DNA extraction. DNA was extracted from 0.5 g of fresh soil following the user guide.

The primer set 515F/806R was used to amplify the V4 region of the bacterial 16S rRNA gene. The polymerase chain reactions (PCRs) were performed in a 50-μL system with a thermal cycle of initial denaturation at 95°C for 10 min; 30 cycles in a series of denaturation at 94°C for 1 min, annealing at 55°C for 1 min, and extension at 72°C for 1 min; and a final extension at 72°C for 10 min. The PCR system consisted of 25 μl PCR premix (TaKaRa Ex Taq), 1 μl forward primer (10 μM), 1 μl reverse primer (10 μM), 1 μl DNA template (20 ng), and 22 μl PCR grade water. After quality checks and purification, the PCR products were sequenced using an Illumina HiSeq 2,500 System.

### Bioinformatic analysis of the high-throughput sequencing data

Bioinformatic analysis of the sequencing data was primarily performed using the VSEARCH package (Rognes et al., 2016) according to the protocols described in previous studies (Liu et al., 2020; Sun et al., 2022b). After removing adapt and primer bases using Cutadapt (Martin, 2011), the paired-end reads were merged; then, the low-quality and chimeric reads were filtered. The clean reads were then denoised using the UNOISE algorithm (version 3), and zOTUs (zero-radius operational taxonomic units) were generated. Taxonomic assignment of each zOTU was performed using SINTAX (Edgar, 2016) against SILVA rRNA database (version 138). The zOTUs assigned as mitochondria or chloroplast were removed; then, the zOTU table was rarefied to 48,000 reads per sample for statistical analysis.

### Determination of microbial P transformation profiles

The functional profiles of the bacterial community were predicted using PICRUSt2 (Phylogenetic Investigation of Communities by Reconstruction of Unobserved Stats; Douglas et al., 2020). Genes involved in P transformation were selected for further analysis.

Soil alkaline phosphatase activity was determined using a Soil Alkaline Phosphatase (S-AKP/ALP) Activity Assay Kit (Beijing Solarbio Science & Technology Co., Ltd., China) following the user’s manual.

The inorganic P dissolution potential of the soil microbial community was measured using an incubation method according to the previous study (Sun et al., 2022b). In short, 1 g of soil was added to 90 ml of sterilized water to make a soil suspension, and 1 ml of the soil suspension was added to a 150 ml conical flask filled with 50 ml sterile PVK liquid medium. Then, the flask was incubated at 28°C (180 rpm) for 5 days. Dissolved P in the medium was measured at 0, 12, 24, 48, 72, 96, and 120 h.

## Statistical analysis

Statistical analysis was performed using R platform (version 4.0.2) according to the previous studies (Sun et al., 2021, 2023a). The significance of the variable difference was checked by Kruskal–Wallis rank-sum test (0.05 level). Non-metric multidimensional scaling (NMDS) based on Bray–Curtis distance was performed to illustrate the variation of the bacterial community under different treatments. Beta-null model deviation was calculated to show the changes in deterministic and stochastic processes in soil microbial community assembly (Tucker et al., 2016; Sun et al., 2020). Pearson’s correlation coefficients between bacterial community and soil properties were calculated using the Mantel test, and the best subset of environmental variables having the maximum (rank) correlation with community dissimilarities was determined using ‘bioenv’ function in Vegan library.

## Results

### Effects of manure treatment on soil properties and crop yield

As shown in Table 1, soil pH showed no significant difference between manure treatments and the control. However, soil pH decreased with a high amount of manure application, and soil pH at 1.00 Po was significantly lower than that at 0.25 Po and 0.50 Po. Soil conductivity (EC) varied little among treatments, except for significantly lower EC in 0.50 Po than in the other treatments. Soil total carbon (TC) was significantly higher in the 0.75 Po and 1.00 Po treatments than in the other treatments. The soil total nitrogen (TN) and soil organic matter (SOM) in the manure treatments were similar to those in the control, but 0.50 Po contained significantly lower TN than the other treatments and significantly lower SOM than 0.75 Po and 1.00 Po. Soil ammonia (NH_4_^+^‑N) and nitrate nitrogen (NO_3_‑N) in 0.25 Po and 0.50 Po were significantly lower than that in the control, while 0.75 Po and 1.00 Po contained similar levels of NH_4_^+^‑N and NO_3_‑N as the control.

**Table 1:** Soil properties under different treatments.

Property	Control	0.25Po	0.50Po	0.75Po	1.00Po
pH	7.82 (0.27)abc	7.81 (0.01)c	7.80 (0.1)bc	7.68 (0.07)ab	7.62 (0.05)a
EC (μs·cm)	318 (17.58)b	292.67 (19.3)ab	264.33 (16.17)a	307.33 (11.37)b	331.33 (22.85)b
TC (%)	2.16 (0.24)b	2.27 (0.25)ab	2.14 (0.12)b	2.54 (0.25)a	2.60 (0.18)a
TN (%)	0.18 (0.02)ab	0.17 (0.02)ab	0.16 (0.02)b	0.21 (0.06)ab	0.20 (0.02)a
SOM (mg·kg^−1^)	1.54 (0.10)abc	1.50 (0.07)bc	1.45 (0.12)c	1.59 (0.02)a	1.57 (0.05)ab
NH_4_^+^‑N (mg·kg^−1^)	1.41 (0.69)b	0.80 (0.28)a	0.75 (0.15)a	1.01 (0.15)ab	1.30 (0.56)ab
NO_3_‑N (mg·kg^−1^)	17.39 (0.23)b	14.03 (2.01)a	12.78 (2.57)a	16.76 (1.67)ab	17.74 (1.41)ab
AP (mg·kg^−1^)	179.79 (20.29)b	188.55 (28.91)b	204.83 (24.42)ab	198.34 (36.8)ab	243.63 (15.95)a
Tomato yield (t·h^−1^)	132.79 (1.21)a	128.63 (4.23)a	133.75 (4.01)a	133.24 (2.91)a	126.52 (7.98)a
Muskmelon yield (t·h^−1^)	85.73 (6.95)a	88.35 (1.36)a	88.35 (2.80)a	93.07 (4.34)a	93.20 (4.90)a

EC, conductivity; TC, total carbon; TN, total nitrogen; SOM, soil organic matter; NH_4_^+^‑N, ammonia nitrogen; NO_3_‑N, nitrate nitrogen; AP, available phosphorus. Data containing the same letter indicate no significant difference between treatments detected by the Kruskal–Wallis rank-sum test (0.05 level).

All the manure treatments contained higher AP than the control, and the AP content increased with a high amount of manure application, but only the increase in AP at 1.00 Po was statistically significant.

Tomato yields did not differ significantly between the control and manure treatments (Table 1), and muskmelon yields increased with a high amount of manure application, but the difference was not significant (Table 1).

### Variation in the bacterial community under different treatments

**Figure 1:**
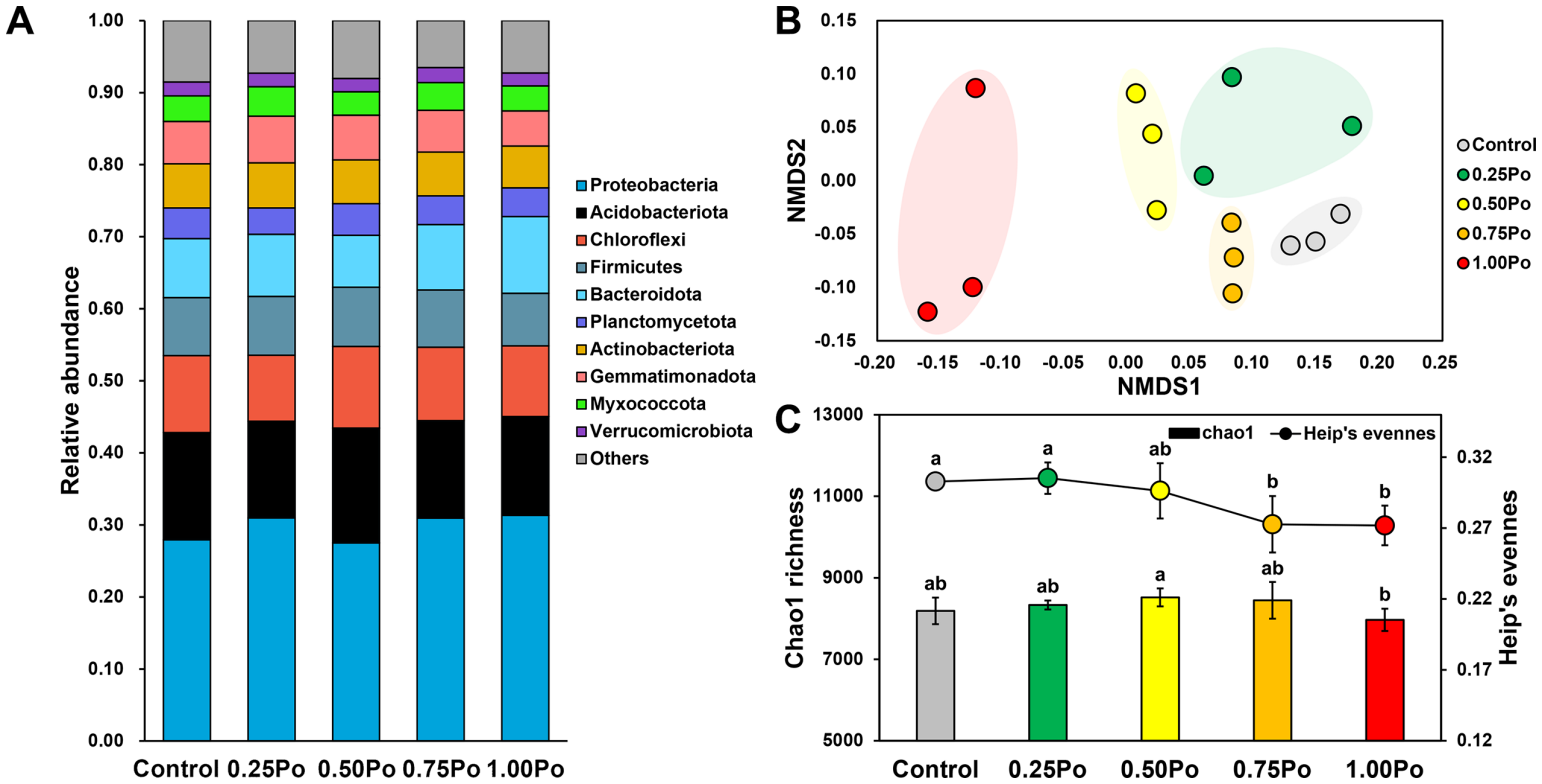
Taxonomic composition of the bacterial community under different treatments **(A)**. NMDS plots show the variation in the bacterial community **(B)**. Chao1 richness (bar plot) and Heip’s evenness (point plot) of the bacterial community under different treatments **(C)**. Bars with different letters (shown above each) are significantly different (*p* < 0.05) as revealed by the Kruskal–Wallis rank-sum test.

The bacterial community in the studied soil was dominated by Proteobacteria, Acidobacteriota, Chloroflexi, Firmicutes, Bacteroidota, Planctomycetota, Actinobacteriota, Gemmatimonadota, Myxococcota, and Verrucomicrobiota, which accounted for 92.47% of the total reads (Figure 1A). The variation in the bacterial community under different treatments was illustrated by a 2D NMDS plot (Figure 1B), in which the samples from different treatments were separated from each other, suggesting that different treatments shaped distinct bacterial communities.

Chao1 richness and Heip’s evenness were calculated to assess the impact of different treatments on bacterial diversity (Figure 1C). The Chao1 richness was not significantly different between manure treatments and the control, but 1.00 Po harbored significantly lower bacterial richness than 0.50 Po. In contrast, Heip’s evenness was lower in manure treatments than in the control, and 0.75 Po and 1.00 Po resulted in significantly lower bacterial evenness than the control.

### Impact of manure treatment on soil bacterial functional profiles involved in P transformation

**Figure 2:**
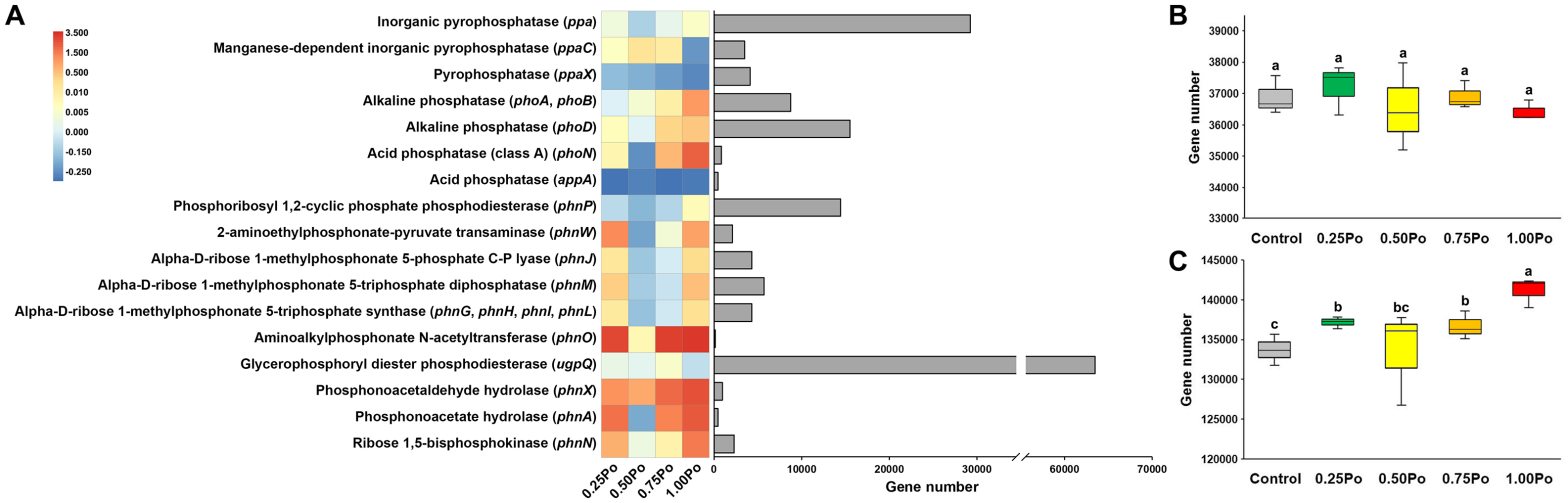
Fold change in abundance of the genes involved in P transformation compared with control (heatmap) and the total abundance of each gene (bar plot; **A**). Total abundance of genes involved in Pi dissolution **(B)** and Po mineralization **(C)**. Boxes with different letters (shown above each) are significantly different (*p* < 0.05) as revealed by the Kruskal–Wallis rank-sum test.

Using PICRUSt2, 20 genes involved in P transformation were predicted (Figure 2A). In general, manure treatment increased the abundance of most of the functional genes, except for that of the *ppaX* and *appA* genes. In addition, the dosage effect of manure on these functional genes was also observed. For example, 0.25 Po, 0.50 Po, and 0.75 Po increased, but 1.00 Po decreased, the abundance of the *ppaC* gene. Po and 1.00 Po increased, but 0.50 Po and 0.75 Po decreased, the abundance of the *phnW* gene. When grouping the genes involved in inorganic P dissolution and organic P mineralization, the total abundance of genes involved in inorganic P dissolution varied little among treatments (Figure 2B). In contrast, the total abundance of genes involved in organic P mineralization was significantly higher in manure treatments (except 0.50 Po) than in the control (Figure 2C).

**Figure 3:**
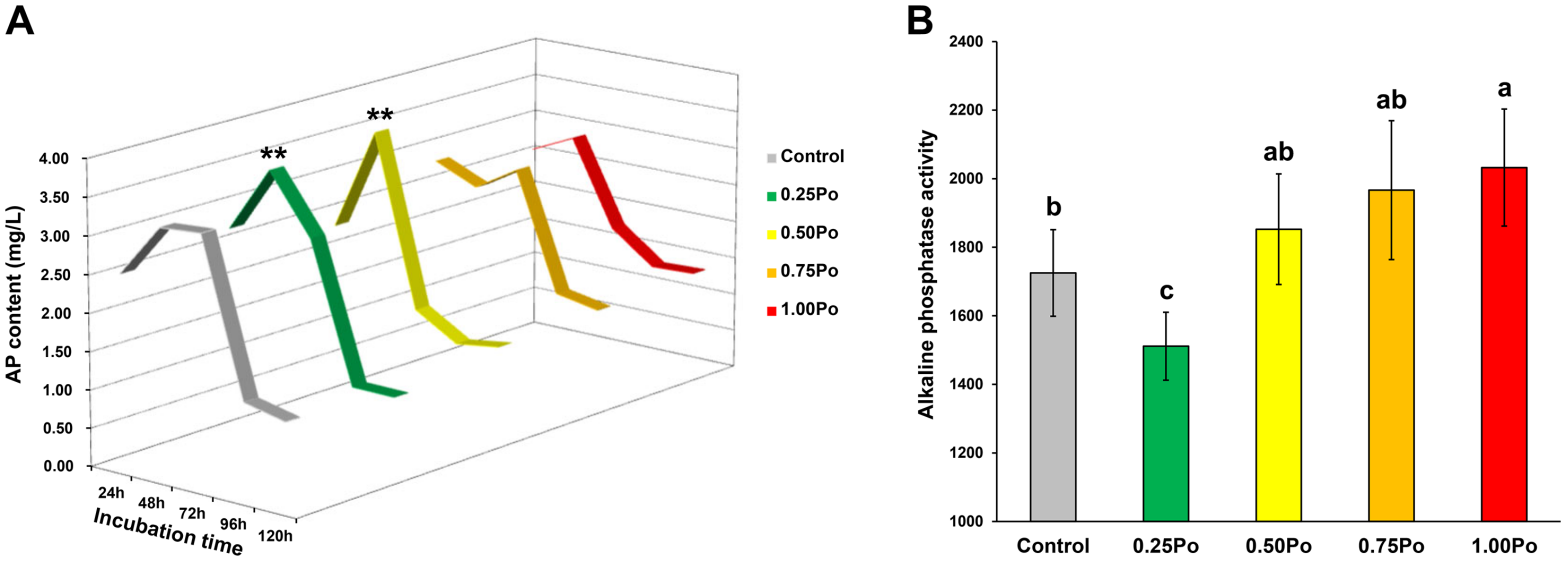
Dynamics of inorganic P solubilization by microbes **(A)** and changes in soil alkaline phosphatase activity **(B)** under different treatments. **Indicates a significant difference compared with the control. Bars with different letters (shown above each) are significantly different (*p* < 0.05) as revealed by the Kruskal–Wallis rank-sum test.

Based on the incubation experiment using calcium phosphate as the sole P resource, the microbial inorganic P dissolution capacity under different treatments was illustrated (Figure 3A). The dynamic changes in dissolved P in the culture showed an approximate bell curve during the whole incubation period. The highest content of dissolved P was observed at 48 h. The 0.75 Po and 1.00 Po treatments showed similar dynamic curves as the control. However, the 0.25 Po and 0.50 Po treatments resulted in significantly higher dissolved P contents at 48 h than the control.

The soil alkaline phosphatase activity in the 0.50 Po and 0.75 Po treatments was similar to that in the control (Figure 3A), but significantly lower and higher soil alkaline phosphatase activity than that in the control was observed in the 0.25 Po and 1.00 Po treatments, respectively (Figure 3B).

### Correlations between soil properties and the bacterial community

Through the Mantel test, the Pearson correlation coefficients between soil properties and the bacterial community were determined (Table 2). The results showed that in all the detected soil properties, TC, TN, and phosphatase activity were significantly correlated with the bacterial community. Soil pH, TC, TN, and AP were the best subsets with the maximum (rank) correlation with the community. The subsets’ correlations with the bacterial community were much higher than the single correlations.

**Table 2:** Pearson’s correlation coefficients between soil properties and bacterial community.

Factors	*r*	*p* value
pH	0.244	0.139
EC	−0.118	0.752
TC	**0.437**	0.015
TN	**0.418**	0.030
SOM	−0.057	0.570
NH_4_	0.069	0.287
NO_3_	−0.198	0.943
AP	0.029	0.375
Apase	**0.175**	0.043
Best subset pH, TC, TN, AP	**0.595**	0.002

Bold numbers indicate significant correlations.

### Microbial community assembly under different manure treatments

**Figure 4:**
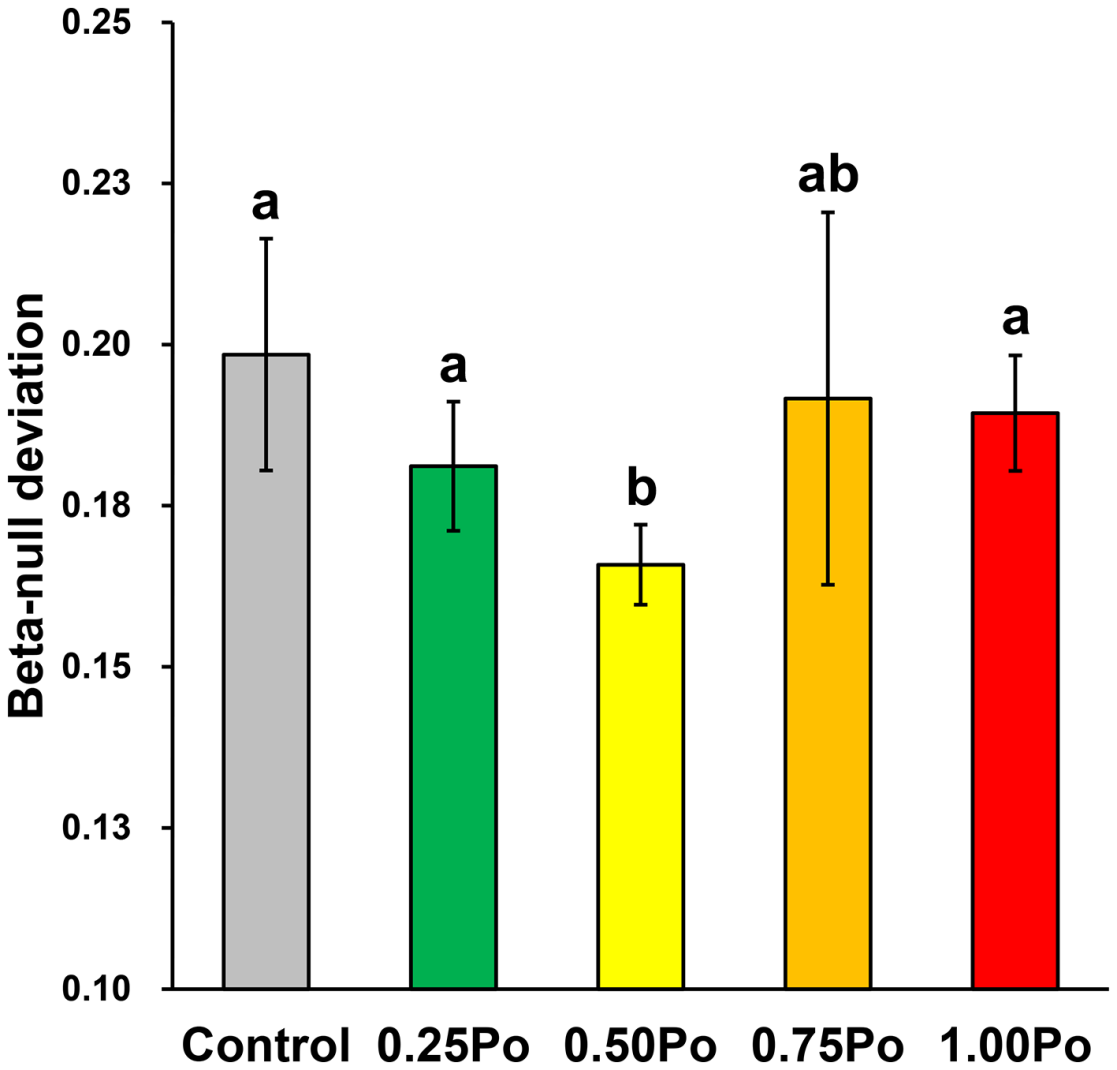
Beta-null deviations of the bacterial community under different treatments. Bars with different letters (shown above each) are significantly different (*p* < 0.05) as revealed by the Kruskal–Wallis rank-sum test.

The positive beta-null deviation revealed the dissimilarity between the detected bacterial community and the randomly assembled community from the null model. In addition, the beta-null deviation in the 0.50 Po treatment was significantly lower than that in the control, while it was similar between the other manure treatments and the control (Figure 4). These results showed that the contribution of deterministic assembly in shaping the bacterial community was significantly lower in the 0.50 Po treatment than in the control, and the 0.25 Po, 0.75 Po, and 1.00 Po treatments had little impact on soil bacterial community assembly.

## Discussion

Greenhouse agriculture is a potential sustainable production mode for future agriculture. However, a high input of resources greatly hinders its development. As a widely sourced organic material, manure is considered a great substitute for mineral fertilizers. Our previous study revealed that substituting mineral P fertilizer with manure increased soil P availability and enhanced microbial P transformation, especially for organic P mineralization (Sun et al., 2022a). In this study, the dosage effects of manure on soil P availability and microbial P transformation were further revealed. Using manure as the sole P resource at the amount of 25, 50, and 75% of the total input P in the control could keep the AP level in soil similar to that in the control, indicating that manure could not only be a good substitute for mineral P fertilizers but also decrease the input of P resources. However, the application of manure as the soil P resource at the same amount of P input in the control significantly increased the soil AP content, reaching 243.63 mg/kg (Table 1). Such a high amount of AP may increase the risk of P leaching.

Soil microbes are the primary drivers of P transformation (Alori et al., 2017; Park et al., 2022). Studies have shown that manure application increases soil P availability through various mechanisms, such as readily inputting AP, releasing native soil P, decreasing the availability of sorption sites (Poblete-Grant et al., 2020), and enhancing soil microbial P transformation capacity (Chen et al., 2022). Here, the dosage effects of manure on soil bacterial community assemblages and P transformation features were also revealed. Although the taxonomy and richness of the bacterial community varied little among the different manure treatments (Figures 1A,C), different manure treatments shaped distinct bacterial community structures (Figure 1B), and a high input of manure significantly decreased community evenness (Figures 1B,C), indicating that the bacterial community assemblage sensitively responded to manure application and that the impact of manure on the bacterial community intensified with increasing manure amount. The dosage effects of manure on the soil bacterial community were also observed in the P transformation capacity. Low inputs of manure, 0.25 Po and 0.50 Po, significantly enhanced microbial inorganic P dissolution (Figure 3A); however, the opposite phenomenon was observed for organic P mineralization, with the lowest soil alkaline phosphatase activity observed in the 0.25 Po treatment and the highest in the 1.00 Po treatment (Figure 3B). These findings revealed the distinct mechanisms of low and high inputs of manure in affecting soil P availability: Low inputs of manure increased AP sourced from inorganic P dissolution, while high inputs of manure increased AP sourced from organic P mineralization. However, this was inconsistent with the results from function prediction (Figures 2B,C), which showed that manure treatment had little impact on the abundance of functional genes involved in inorganic P dissolution but increased that involved in organic P mineralization. This result showed the uncoupling between microbial potential function and apparent function, which was also observed in many other ecosystems (Torsvik and Øvreås, 2002). This is largely associated with the high functional redundancy of the microbial community (Louca et al., 2018), and the niche separation and taxon-specific responses of the microbial taxa driving the same processes of environmental changes that have been revealed in our previous studies (Sun et al., 2019, 2021). The results from this study indicate that soil P transformation may greatly depend on part of the functional groups, and identifying the core functional microbiome and their community assembly mechanisms would promote the development of microbial regulation techniques to enhance soil P availability.

Understanding the mechanisms of agricultural practices in shaping the soil microbial community is key for the development of sustainable agriculture. Manure application can shape soil microbial community composition and diversity by changing soil environmental properties (Shen et al., 2011; Dai et al., 2020), introducing exogenous taxa (Sun et al., 2016, 2020), altering microbial interactions (Sun et al., 2020), and so on. In this study, the dosage effect of manure on the soil bacterial community may be primarily associated with the different impacts of manure treatments on soil environmental conditions. The soil bacterial community was significantly correlated with two single properties: soil carbon and nitrogen; however, the subset containing pH, TC, TN, and AP showed a much higher correlation with the bacterial community (Table 2), indicating that the soil bacterial community was impacted by multiple factors. As different microbial taxa have distinct environmental preferences (Sun et al., 2021), they showed different responses to environmental changes. In addition, the dosage effect of manure on the soil microbial community may also be associated with different amounts of input carbon. Manure contains various types and high amounts of organic carbon. The input of manure would largely change the supply of carbon resources, thus resulting in a source filter on the soil microbial community. Therefore, the growth and competitiveness of the taxa targeting the carbon resources from manure would improve, resulting in the enhanced dominance of these taxa, while other taxa would become less dominant. If this filter effect is excessively high, the taxa with low competitiveness would become extinct, thus resulting in the loss of soil microbial diversity. In general, this filter effect increases with the input amount of manure. Thus, a significant impact on bacterial diversity was observed in the 0.75 Po and 1.00 Po treatments (Figure 1C). The loss of soil microbial diversity by manure application has also been observed in other systems (Sun et al., 2020). These results emphasized the importance of a reasonable application amount of manure in maintaining soil microbial diversity. It is also indicated that substituting mineral P with manure at an amount of 50% P input as conventional fertilization is more beneficial for the soil bacterial community without diverse impacts on soil P availability and crop production. This is further supported by the results of beta-null deviation (Figure 4). The amount of 0.50 Po significantly lowered the contribution of deterministic processes in structuring bacterial community assembly, indicating that the 0.50 Po treatment weakened the niche filter effects on the soil bacterial community, thus increasing diversity. In addition, manure contains types of exogenous bacteria, which would immigrate into the soil by fertilization. Studies have found that manure-sourced microbes are also a key factor influencing soil microbial diversity and community assemblage (Sun et al., 2016, 2020). How exogenous microbes impact soil microbial functions should be paid more attention in further studies.

## Conclusion

Manure is considered a favorable substitute for mineral P fertilizers. This study revealed the dosage effect of manure application on soil P availability, bacterial community assemblage, and P transformation functional features in greenhouse agriculture. Without mineral P fertilizers, manure application could maintain soil P availability as traditional fertilization, and a high input of manure significantly increased the soil AP content. However, low and high inputs of manure showed different pathways in impacting soil P availability. A low input of manure enhanced soil microbial Pi dissolution, while a high input of manure enhanced soil microbial Po mineralization. Different amounts of manure input shaped distinct soil bacterial communities, which were closely correlated with the changes in soil pH, TC, TN, and AP. High input of manure significantly lowered bacterial evenness and showed an adverse effect on bacterial richness. Under the comprehensive consideration of the impact of manure on soil P availability, crop production, and soil bacterial diversity, the application of manure as the sole P source at 50% P input as traditional fertilization is reasonable in the studied greenhouse agriculture.

## Data availability statement

The data presented in the study are deposited in the European Nucleotide Archive (ENA) repository, accession number PRJEB60932.

## Author contributions

JN and RS designed the experiment. LZ, XL, ZZ, KL, and FW performed laboratory measurement. LZ and RS performed data analysis. LZ, RS, and CZ wrote the study. All authors contributed to the article and approved the submitted version.

## Funding

This study was supported by the Key Science and Technology Project of Anhui Province (202103a06020012) and S&T Program of Hebei (21326904D).

## Conflict of interest

The authors declare that the research was conducted in the absence of any commercial or financial relationships that could be construed as a potential conflict of interest.

## Publisher’s note

All claims expressed in this article are solely those of the authors and do not necessarily represent those of their affiliated organizations, or those of the publisher, the editors and the reviewers. Any product that may be evaluated in this article, or claim that may be made by its manufacturer, is not guaranteed or endorsed by the publisher.
